# Cryo-EM structures of human p97 double hexamer capture potentiated ATPase-competent state

**DOI:** 10.1038/s41421-022-00379-1

**Published:** 2022-02-22

**Authors:** Haishan Gao, Faxiang Li, Zhejian Ji, Zhubing Shi, Yang Li, Hongtao Yu

**Affiliations:** 1 School of Life Sciences, Westlake University, Hangzhou, Zhejiang China; 2 Westlake Laboratory of Life Sciences and Biomedicine, Hangzhou, Zhejiang China; 3 Department of Pharmacology, University of Texas Southwestern Medical Center, Dallas, TX USA; 4 Center for Medical Genetics, School of Life Sciences, Central South University, Changsha, Hunan China; 5 Howard Hughes Medical Institute, Department of Cell Biology, Harvard Medical School, Boston, MA USA; 6 Department of Biophysics, University of Texas Southwestern Medical Center, Dallas, TX USA

**Keywords:** Cryoelectron microscopy, Ubiquitylation

## Abstract

The conserved ATPase p97 (Cdc48 in yeast) and adaptors mediate diverse cellular processes through unfolding polyubiquitinated proteins and extracting them from macromolecular assemblies and membranes for disaggregation and degradation. The tandem ATPase domains (D1 and D2) of the p97/Cdc48 hexamer form stacked rings. p97/Cdc48 can unfold substrates by threading them through the central pore. The pore loops critical for substrate unfolding are, however, not well-ordered in substrate-free p97/Cdc48 conformations. How p97/Cdc48 organizes its pore loops for substrate engagement is unclear. Here we show that p97/Cdc48 can form double hexamers (DH) connected through the D2 ring. Cryo-EM structures of p97 DH reveal an ATPase-competent conformation with ordered pore loops. The C-terminal extension (CTE) links neighboring D2s in each hexamer and expands the central pore of the D2 ring. Mutations of Cdc48 CTE abolish substrate unfolding. We propose that the p97/Cdc48 DH captures a potentiated state poised for substrate engagement.

## Introduction

The highly conserved molecular chaperone p97, also called valosin-containing protein in humans and Cdc48 in the budding yeast, is a member of the ATPases associated with diverse cellular activities (AAA) family of ATPases^1,2^. As a master molecular machine in protein quality control, p97/Cdc48 is critical for cellular protein homeostasis^3,4^. In complex with a variety of cofactors and adaptors, p97/Cdc48 acts as a segregase or unfoldase that extracts polyubiquitinated proteins from membranes, chromatin, macromolecular complexes, and misfolded protein aggregates for subsequent degradation or remodeling^1,3,5–7^. As such, it regulates diverse cellular processes, including endoplasmic reticulum-associated degradation, mitochondrial-associated degradation, membrane fusion, and DNA replication and repair^1–3^. Mutations of p97 have been linked to human neurodegenerative proteopathy diseases^8,9^. As p97 is overexpressed in certain cancers, there is ongoing interest in developing chemical inhibitors of p97 and its adaptors for cancer therapy^4,10^.

p97/Cdc48 forms a ring-shaped homo-hexamer, with each protomer containing an N-terminal (N) domain, two tandem ATPase (D1 and D2) domains, and a C-terminal extension (CTE)^2,11–14^. The N domain interacts with various substrate-binding adaptors, including the Ufd1–Npl4 complex (UN) that recognizes polyubiquitin chains and the DNA-binding metalloprotease Spartan (SPRTN)^15–19^. The D1 and D2 domains form two stacked hexameric rings with a central pore^12,14,20–22^. Both D1 and D2 domains can hydrolyze ATP, but only the ATPase activity of D2 domains is critical for substrate unfolding^23–26^. The N domain can occupy positions that are coplanar to the D1 ring (down conformation) or above the D1 ring (up conformation)^20,27^. ATP binding at the D1 ATPase site and cofactor binding by the N domain are thought to favor the up conformation^20,23^. The CTE mediates the binding of additional p97/Cdc48 cofactors^1,11,28^. Whether the CTE also has direct roles in ATP hydrolysis and substrate unfolding is unclear.

Recent biochemical and structural analyses of the yeast Cdc48–UN complex in the presence of polyubiquitinated substrates have provided key insight into p97/Cdc48-dependent substrate unfolding^7,17,29,30^. Binding of polyubiquitinated substrates to Cdc48–UN triggers the unfolding of a ubiquitin molecule of the polyubiquitin chain^29^. The unfolded ubiquitin polypeptide is threaded into the central pore of Cdc48 and reaches the D2 ring^29^. The pore loops of the D2 domain adopt a staircase arrangement, and the conserved aromatic residues located in the pore loops engage the unfolded ubiquitin^29^. ATP hydrolysis then drives the movement of pore loops and continuously unfolds and moves the unfolded ubiquitin and ultimately the ubiquitinated substrate through the central pore via a conveyer belt-like mechanism^29^.

The unfolding of the initial ubiquitin molecule that initiates substrate unfolding by Cdc48–UN does not require ATP hydrolysis by Cdc48^29^. This finding suggests that the binding energy between the ubiquitinated substrate and Cdc48–UN suffices to unfold ubiquitin, a stably folded protein. Because Cdc48 is required for this unfolding event, the engagement between the unfolded ubiquitin and the pore loops of Cdc48 contributes to ubiquitin unfolding. Yet, the pore loops of p97/Cdc48 are disordered in the absence of substrates and occlude the central pore. Whether and how the pore loops become ordered before substrate engagement are unclear.

While the major pool of human p97 forms a hexamer, a minor population of p97 is known to exist as a double hexamer (DH), which is thought to be catalytically inactive^27,31–36^. Here, we show that both human p97 and yeast Cdc48 can form DHs in vitro, which are functional ATPases. Cdc48 DH is capable of unfolding a polyubiquitinated substrate. We have further determined the structures of human p97 DH in both the ADP- and ATP-bound states using cryo-electron microscopy (cryo-EM). p97 DH consists of two single hexamers (SH) connected by their D2 rings. The C-terminal helix (α9) and its preceding loop constitute the major inter-SH contacts, resulting in a tail-to-tail stacked DH. Furthermore, the CTE becomes ordered in the DH and wraps around the adjacent protomer in the same SH. Deletion of α9 or CTE disrupts the p97 DH and decreases its overall ATPase activity. The α9 and CTE of Cdc48 are required for substrate unfolding. Thus, our study reveals a direct involvement of the CTE in inter-protomer communication and substrate unfolding.

## Results

### Architecture of human p97 double hexamer

**Fig. 1:**
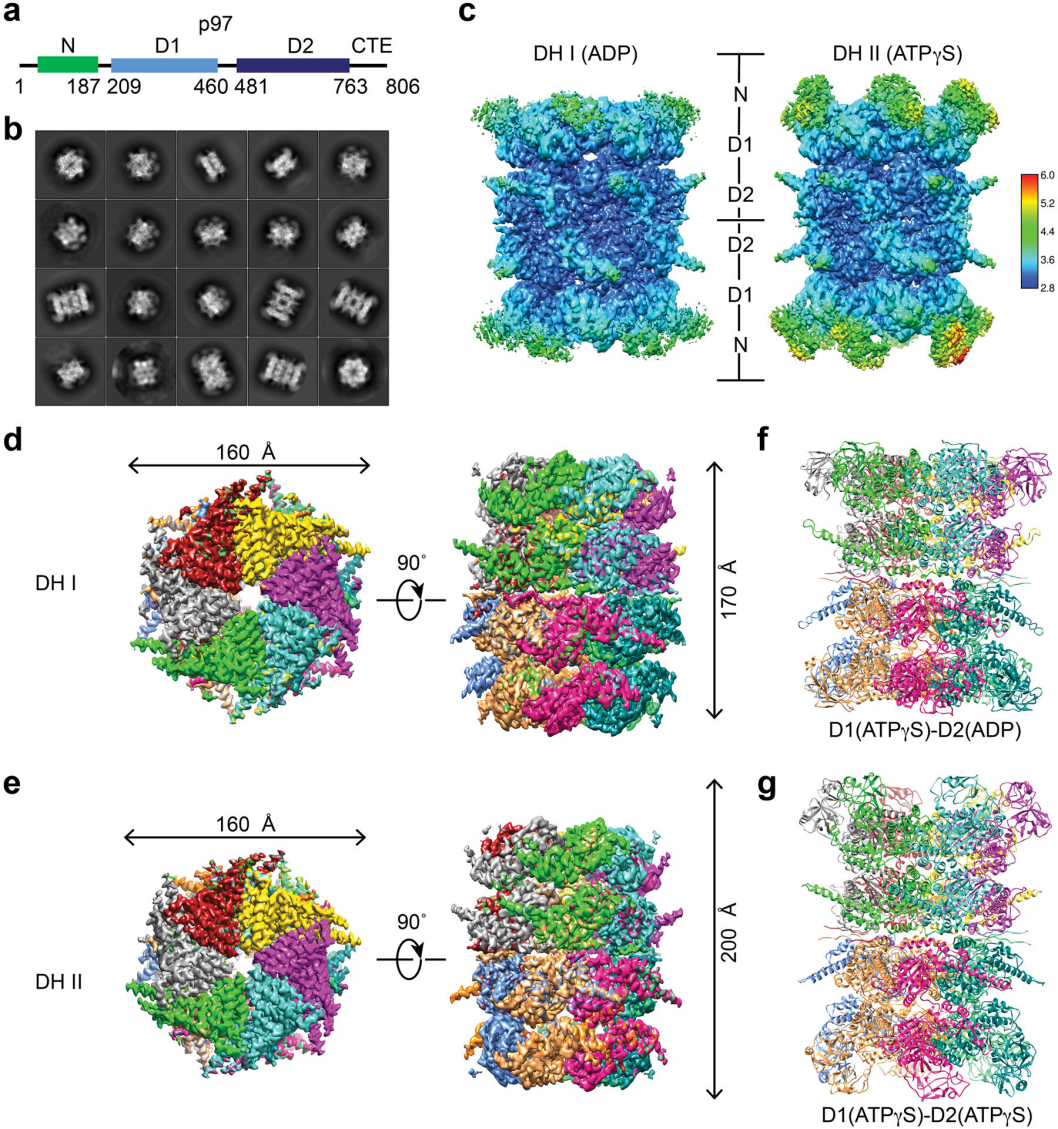
Cryo-EM structures of human p97 double hexamer (DH). **a** Domains and motifs of human (Hs) p97. **b** Representative 2D class averages of human p97-SPRTN complex. **c** Local resolution maps of human p97 DH conformers I (left) and II (right). 3D reconstructions of p97 DH I (**d**) and DH II (**e**) in top (left) and side (right) views. Each p97 protomer is colored differently. Ribbon diagrams of the p97 DH I (**f**) and DH II (**g**). The identities of the bound nucleotides are indicated.

The metalloprotease Spartan (SPRTN, also called DVC1) maintains genome stability by cleaving DNA-protein crosslinks^16,18,19^. SPRTN contains an SHP motif, which interacts with p97 (Fig. 1a and Supplementary Fig. S1a, b), and recruits it to chromatin to remove the translesion synthesis DNA polymerase η (Pol η) after lesion bypass^18,19^. Because of our interest in SPRTN, we purified the recombinant human p97–SPRTN complex and analyzed it by negative-stain EM and single-particle cryo-EM (Supplementary Fig. S1c–e). Subsequent 2D classifications revealed that, in addition to the well-characterized p97 SH, certain 2D classes clearly represented p97 DH (Fig. 1b; Supplementary Fig. S1e, f). Using the same dataset, we determined five structures of p97 in total: three SH conformations (I, II, and III), and two DH conformations (I and II) (Fig. 1c–g; Supplementary Figs. S1g–i, S2, S3a–i, and Table S1). The cryo-EM density maps were of sufficient quality to build models of all parts of the protein except the flexible N domain. We focused on the structure analysis of p97 DH, as it had not been well studied.

The 2D classification indicated the presence of a C_6_ symmetry along the vertical axis and a two-fold rotational symmetry along the horizontal axis of p97 DH (Fig. 1b). We imposed D_6_ symmetry on both DH conformers I and II and determined their structures to resolutions of 3.15 and 3.32 Å, respectively, according to the Fourier shell correlation (FSC) = 0.143 ‘gold standard’ criterion (Supplementary Fig. S2a–d). Local map resolutions measured for both DH conformations by ResMap^37^ indicate that the density is weakest in the N domain and strongest in the stacked rings of D1 and D2 ATPase domains (Fig. 1c). The D2 domains have a resolution of about 3.0 Å. The nucleotides are well resolved in both p97 SH and DH (Supplementary Figs. S2e–h, S3c, f, i). The D1 domains of both DH I and II conformations are in the ATP-bound state. The D2 domains in DH I are bound to ADP whereas those in DH II bind ATP. The N domains of DH I are in the down conformation while those in DH II are in the up conformation (Fig. 1c, f, g), despite the fact that the D1 domains of both DH I and II are in the ATP-bound state. Thus, the up and down conformations of the N domains are not necessarily coupled to specific nucleotide states in the D1 domain^13,20,23,38–40^.

The p97 DH consists of two identical single hexamers engaged in a tail-to-tail manner, with the two D2 rings directly contacting each other (Fig. 1c–g). This arrangement is different from the head-to-head arrangement of the MCM2-7 double hexamer^41^. The subunit positions are staggered between the two single hexamers in p97 DH. The centers of mass of the six subunits in one hexamer are related to those in the other by a 30˚ rotation along the axis of the central pore (Fig. 1c–g; Supplementary Fig. S2i). We did not observe extra density that could belong to SPRTN in either DH conformer, presumably due to conformational flexibilities between the N and D1 domains of p97 and between the SHP motif and the protease domain of SPRTN.

### p97/Cdc48 DH is an active ATPase

**Fig. 2:**
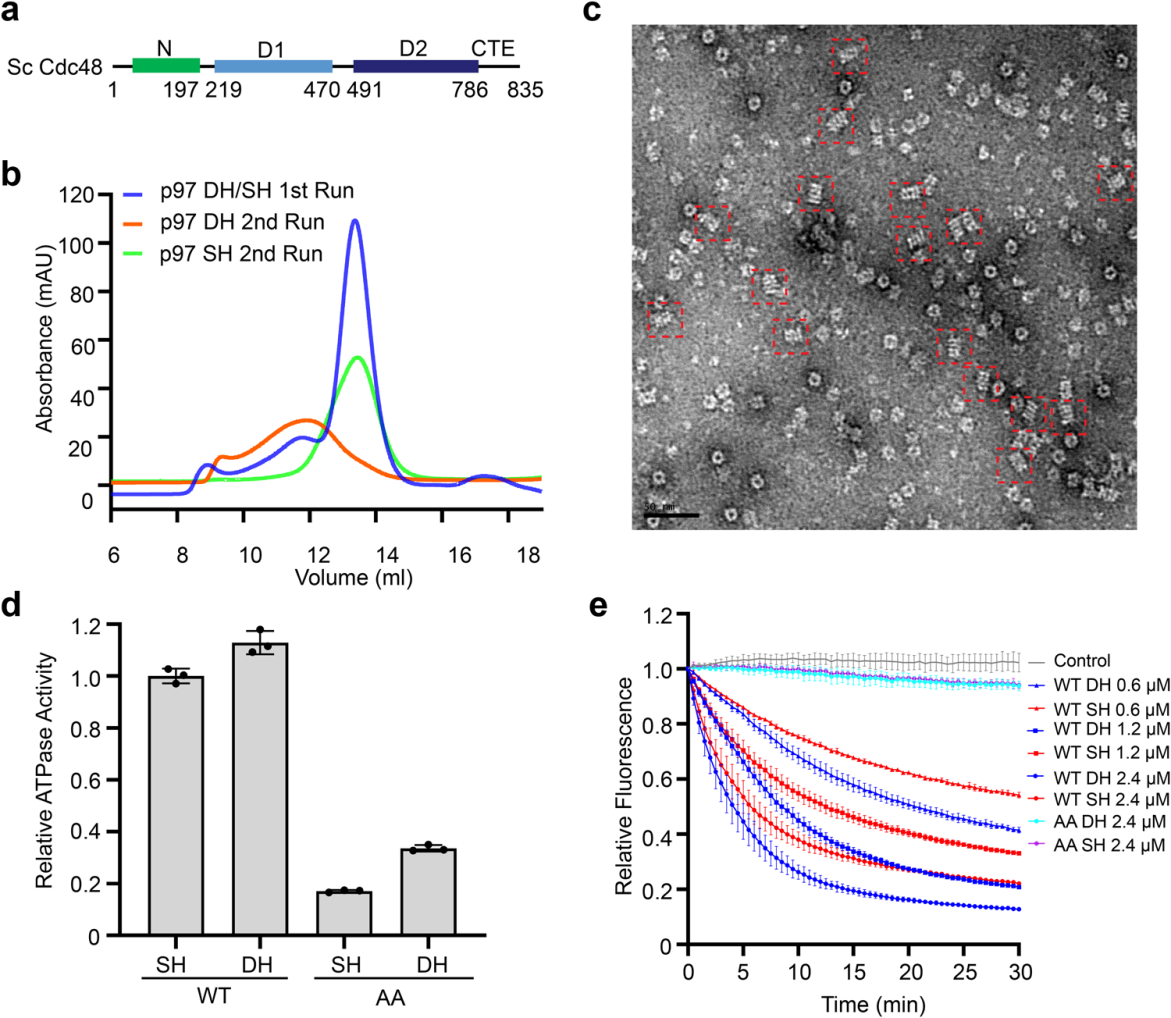
p97/Cdc48 double hexamers (DHs) are active ATPases. **a** Domains and motifs of *Saccharomyces cerevisiae* (Sc) Cdc48. **b** Gel filtration profiles of human p97 on a Superose 6 column. **c** Representative negative stain EM micrograph of human p97 DH. DHs in side views are boxed by red dashed squares. **d** Relative ATPase activities of the indicated human p97 DHs and single hexamers (SHs) at the protomer concentration of 120 nM. WT, wild type; AA, E305A/E578A, p97 mutant deficient in ATPase activities of both D1 and D2 domains. The activities were normalized to that of WT SH. Data are shown as means ± SEM (*n* = 3 independent experiments). **e** Substrate unfolding assay by the indicated Sc Cdc48 DHs and SHs at varying protomer concentrations. WT, wild type; AA, E315A/E588A, Cdc48 mutant deficient in ATPase activities of both D1 and D2 domains. Data are shown as means ± SEM (*n* = 3 independent experiments).

Higher order oligomers of p97 in the absence of cofactors have been reported previously^27,31–36^. In addition, p97 DH with a similar tail-to-tail arrangement of two single hexamers can form in p97 crystals through crystal packing interactions^42^. To confirm that p97 could indeed form DH in the absence of SPRTN, we purified recombinant human p97 with a C-terminal His_6_ tag. In the absence or presence of different nucleotides, the major population of p97 expectedly fractionated as an SH during gel filtration chromatography, while a minor population fractionated as a higher order oligomer (Fig. 2a, b; Supplementary Fig. S4a). Negative stain EM images of this high-order oligomer population contained many particles with a four-layer architecture that closely resembled the p97 DH in sideview (Fig. 2c). When the two populations of p97 were separately pooled and fractionated again, the SH pool fractionated exclusively as a single hexamer (Fig. 2b). Likewise, the DH stayed as a double hexamer (Fig. 2b). Thus, the formation of p97 DH does not require the binding of SPRTN or other cofactors. The SH and DH pools are not at fast equilibrium in vitro and can be stably isolated.

A pool of p97 expressed in human HEK293 cells also existed as DH, as revealed by negative stain EM and cryo-EM (Supplementary Fig. S4b–d). Affinity capture of endogenous human p97 with small-molecule ligand bait reveals the presence of both SH and DH populations^36^. A recent study also reported the cryo-EM structure of the p97 DH^43^. Gel filtration profiles and negative stain EM studies indicated that yeast Cdc48 also had both SH and DH conformations (Supplementary Fig. S4e, f). The ability to form SH and DH is thus a conserved feature of p97/Cdc48.

We next measured the ATPase activities of p97/Cdc48 SH and DH. In contrast to a previous report claiming that human p97 DH was inactive in hydrolyzing ATP^27^, we showed that both p97/Cdc48 SH and DH were active in ATP hydrolysis with comparable activities (Fig. 2d; Supplementary Fig. 4g, h). As controls, the p97/Cdc48 mutants with the Walker B motif of both D1 and D2 domains mutated (p97 E305A/E578A; Cdc48 E315A/E588A) of either SH or DH had only basal ATPase activities (Fig. 2d; Supplementary Fig. S4g, h).

A substrate unfolding assay for yeast Cdc48 using the polyubiquitinated fluorescent protein mEos3.2 as the substrate had been established^7,17,29,44^. After being irradiated with near-UV light, the polyubiquitinated mEos3.2 protein undergoes peptide backbone cleavage and produces two physically associated fragments that retain fluorescence^7,17,29^. In the presence of the UN adaptor complex and ATP, Cdc48 unfolds the cleaved mEos3.2 and separates the two mEos3.2 fragments, causing the irreversible loss of fluorescence^7,17,29^. Using this in vitro assay, we showed that both Cdc48 SH and DH, but not their ATP-deficient mutants, efficiently unfolded the polyubiquitinated substrate mEos3.2, as evidenced by the time-dependent decrease of the fluorescence signal (Fig. 2e). Therefore, these results indicated that the p97/Cdc48 DH is an active ATPase, and Cdc48 DH can efficiently unfold a ubiquitinated substrate in vitro. We also attempted the substrate unfolding assay for human p97 and its substrate adaptor, but failed to observe substrate unfolding, thus preventing us from comparing the substrate unfolding activities of p97 SH and DH.

### The dimerization interface of p97 DH

**Fig. 3:**
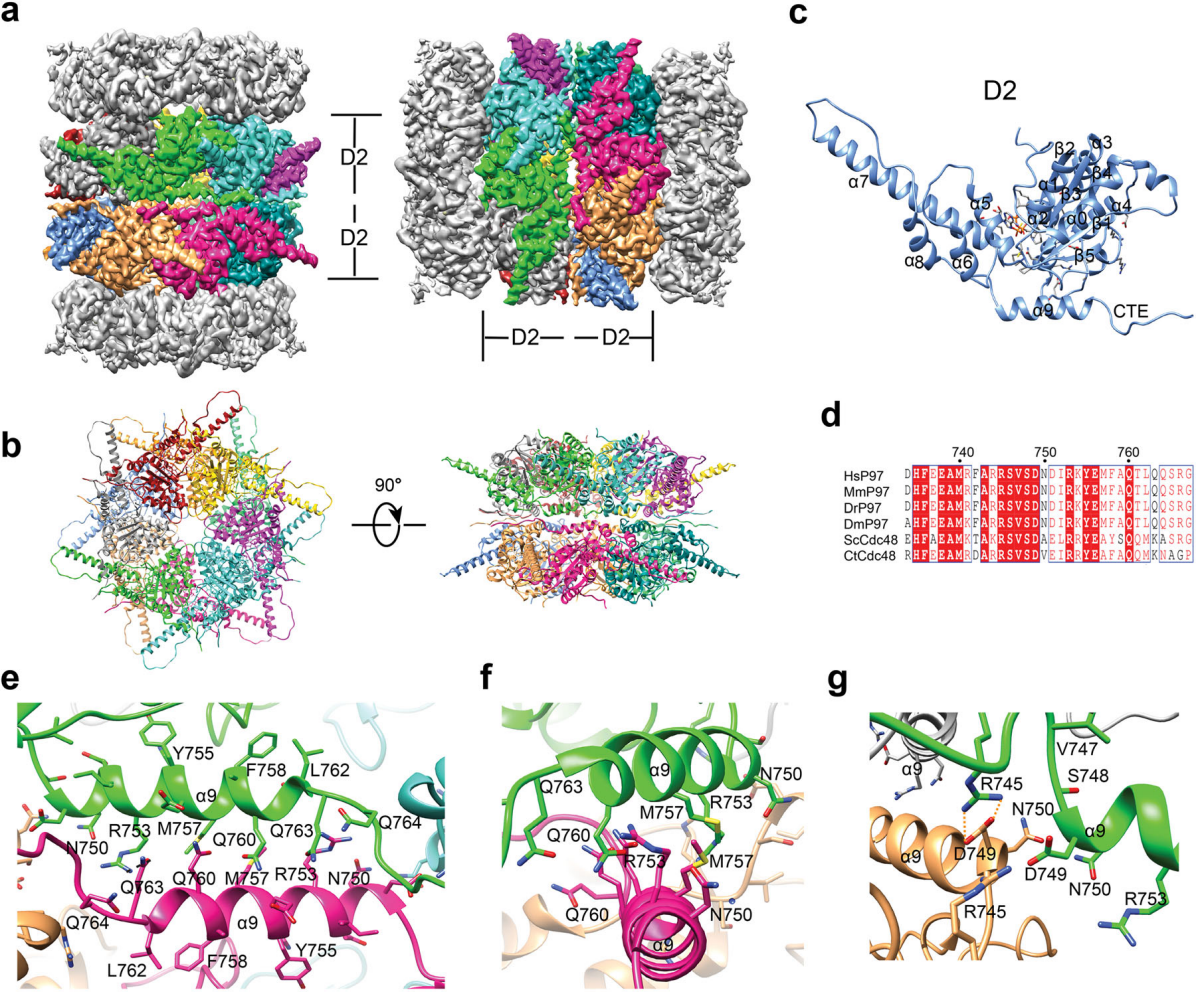
Dimerization of human p97 single hexamer. **a** Cryo-EM map of p97 DH conformer II in two orientations and with the D2 domain of each protomer colored differently. **b** Ribbon diagram of the D2 ring in p97 DH II in top (left) and side (right) views. **c** Ribbon diagram of a single D2 domain in p97 DH II. **d** Sequence alignment of the inter-hexamer dimerization interface residues in p97 DH. Hs, *Homo sapiens*; Mm, *Mus musculus*; Dr, *Danio rerio*; Dm, *Drosophila melanogaster*; Sc, *Saccharomyces cerevisiae*; Ct, *Chaetomium thermophilum*. The sequences were aligned using Clustal Omega and numbered according to human p97. **e***–***g** Interactions at the inter-hexamer dimerization interface of p97 DH II. The indicated residues are shown as sticks. The electrostatic interactions between R745 and D749 are indicated by dashed lines.

In p97 DH, the tail-to-tail stacking of the two SHs is mediated by their D2 rings. This interface is extensive, with a buried surface area of about 20,000 Å^2^. The main interactions between each D2 pair involve the α9 helix and the α8–α9 linker (Fig. 3). The α9 helix from one SH pack against α9 from the other in an anti-parallel manner, forming both hydrophobic and polar interactions (Fig. 3e–g; Supplementary Fig. S2j). R745 located in the α8–α9 linker of one SH forms a salt bridge with D749 at the N-terminal end of α9 from the other SH (Fig. 3g). Both R745 and D749 are highly conserved in p97/Cdc48 proteins from different species (Fig. 3d). Because of the 6-fold symmetry, there are six equivalent α9–α9 interactions and conserved RD (R745-D749) pairs, which make up the SH–SH dimerization interface. Collectively, these interactions connect two SHs to form the DH.

The α9 helix is the major structural element at the dimerization interface. It is located in the vicinity of the D2 ATPase active site (Supplementary Fig. S5). For example, Y755 in the α9 helix directly interacts with the D2 ATPase sensor N624. The dimerization of SHs alters the conformation of the α9 helix in subtle ways, including side chain configurations of α9 residues. Thus, dimerization may influence the D2 ATPase activity of p97.

### Inter-subunit interactions in the D2 ring mediated by the CTE

**Fig. 4:**
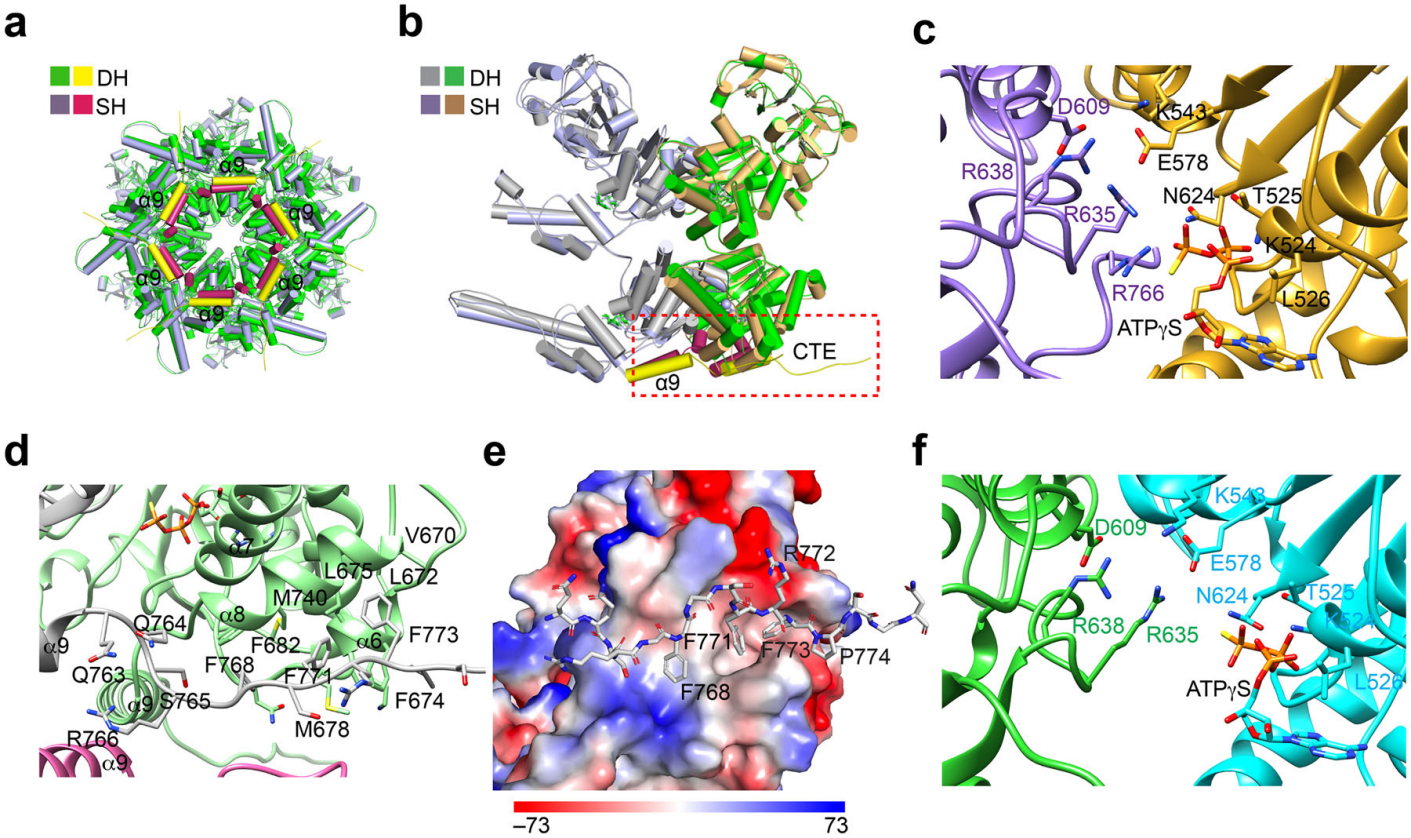
The C-terminal extension (CTE) tethers the D2 ring in p97 double hexamer (DH). **a** Superimposition of ATPγS-bound SH conformer I and the hexamer in DH conformer II. The ɑ9 helices of SH and DH are colored yellow and red, respectively. Other structural elements of SH and DH are colored light blue and green. **b** Superimposition of two adjacent protomers from SH and DH. The two protomers from DH are colored gray and green, while the SH protomers are colored light blue and gold. ɑ9 and CTE are boxed with red dashed lines. **c** The ATP-binding pocket of the D2 ring in SH conformer I. **d** Interactions involving the CTE in the DH. **e** Surface diagram of the CTE-binding site colored by electrostatic potential (red, negative; blue, positive; white, neutral). The CTE is shown as sticks. **f** The ATP-binding pocket of the D2 ring in DH conformer II.

The CTE of p97 is conserved and predicted to be unstructured and flexible^1,2,11,12,14,21,28^. In most published p97 structures, the CTE segment C-terminal to residue Q764 is disordered and invisible^20,21,23,35,42^. In two reported ATPγS-bound p97 structures (5C18 and 5FTN) and our SH conformer I, six additional residues (_765_SRGFGS_770_) were resolved (Fig. 4a, b; Supplementary Fig. S6a). They bind to the cleft formed between the ATPase and lid domains of the adjacent subunit. In particular, the conserved R766 coordinates the γ-phosphate group of ATPγS directly and prevents the arginine finger residue R635 of the adjacent subunit from contacting ATPγS and the active-site residue E578 (Fig. 4c), resulting in an inactive/pre-activated conformation in the D2 ring in the SH^20,23^.

In our structures of p97 DH, a longer segment of the CTE (till residue G776) has clearly visible density and can be modeled with high confidence (Fig. 4a, b, d; Supplementary Fig. S6b). The CTE from one subunit wraps around the adjacent subunit. The three conserved phenylalanine residues (F768/F771/F773; termed the 3F motif) in the CTE insert into the hydrophobic cleft formed by α6 and α8 from the adjacent subunit, with a buried surface of about 1200 Å^2^ (Fig. 4d, e; Supplementary Fig. S6b). Most residues that form the hydrophobic α6–α8 cleft are conserved (Supplementary Fig. S6c) and interact extensively with the sidechains of the 3F motif.

The nucleotides are well resolved in p97 DH (Supplementary Fig. S2e–h). In both DH conformers, the D1 ring is in an ATPase-activated conformation, and the ATP-binding mode is essentially identical to that of SH conformer I (Supplementary Fig. S6d). The nucleotide-binding mode of the D2 ring in the DH is different from that in the SH. Notably, R766 in the CTE no longer inserts into the ATP-binding pocket of the adjacent protomer as seen in the SH (Fig. 4f). Instead, the arginine finger residue R635 from the adjacent protomer contacts the nucleotide and the active-site residue E578, producing an active ATPase configuration. Thus, the CTE not only stabilizes the D2 ring, but also mediates inter-subunit signaling within the D2 ring.

### Ordered pore loops in p97 DH

**Fig. 5:**
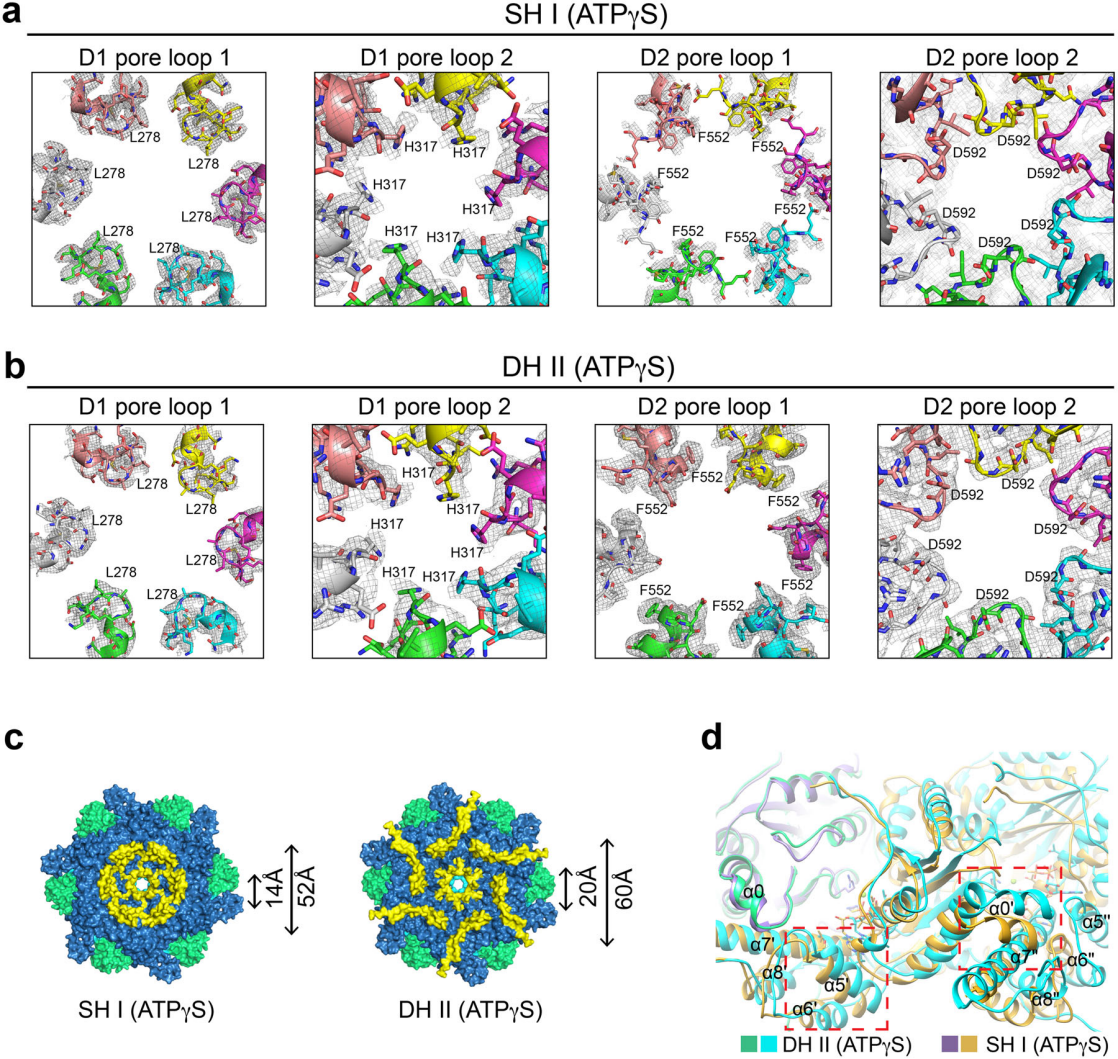
Ordered pore loops in p97 double hexamer (DH). Cryo-EM density of the pore loops in D1 and D2 rings of p97 SH conformer I (**a**) and DH conformer II (**b**). The contour levels for the SH and DH are set at 0.010 (2σ) and 0.020 (5σ), respectively. **c** Surface diagram of SH I (left) and one hexamer of DH II (right) in bottom view. The N, D1, and D2 domains are colored green, cyan, and blue, respectively. ɑ9 and the D2 pore loop 2 are colored yellow. The diameters of the central pore and the D2 ring are indicated. **d** Superimposition of two adjacent protomers of SH I and DH II. Structural elements exhibiting conformational changes are boxed with red dashed lines.

The pore loops of the D2 domain are relatively flexible and not well-resolved in p97 SH conformers I and II, which represent ATPase-inactivated states (Fig. 5a; Supplementary Fig. S7a). The pore loops in both D1 and D2 rings in the DH conformers are ordered and have stronger densities (Fig. 5b; Supplementary Fig. S7b). The D2 ring in p97 DH also has a substantially wider pore than that in the SH (Fig. 5c), likely stemming from differences in inter-subunit interactions in the SH and DH (Fig. 5d). The pore loops of D1 and D2 rings in the substrate-engaged Cdc48 constitute a staircase to translocate and unfold the substrate^29,30^ (Supplementary Fig. S8a, b). The ordered pore loops along the central tunnel of our substrate-free p97 DH arrange in a symmetric and planar manner, suggesting that they are in a resting conformation ready for substrate engagement and further processing (Supplementary Fig. S8c). Structural comparison of single protomers from p97 DH and substrate-bound Cdc48 (PDB code: 6OPC)^30^ shows that either D1 or D2 in each protomer could align well (Supplementary Fig. S8d,e). Substrate binding might induce relative rotations between D1 and D2 domains, which are linked by the D1–D2 flexible linker, producing the staircase configuration of the pore loops in the substrate-engaged state^13,20,29,30,38^.

### Requirement for ɑ9 and the CTE in stabilization and ATPase activity of the D2 ring

**Fig. 6:**
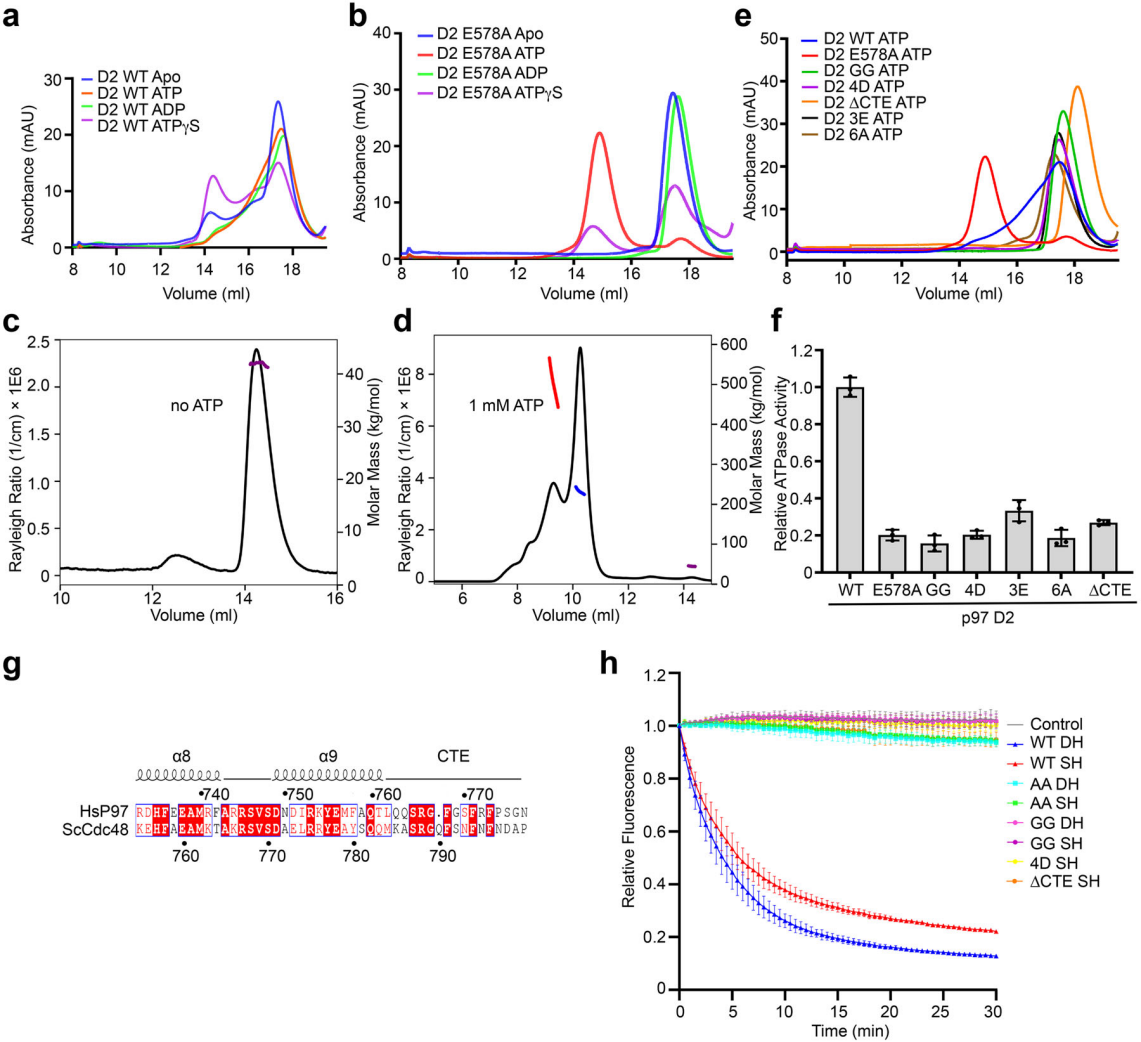
ɑ9 and CTE are required for ATPase activity and substrate unfolding. **a** Gel filtration profiles of the isolated p97 D2 domain (residues 463–806; WT) with different nucleotides on the Superose 6 column. **b** Gel filtration profiles of the p97 D2 ATPase-deficient E578A mutant domain with different nucleotides on the Superose 6 column. Size exclusion multi-angle light scattering (SEC-MALS) profiles of p97 D2 E578A without (**c**) or with (**d**) ATP on the Superdex200 column. **e** Gel filtration profiles of p97 D2 and the indicated mutants with ATP on the Superose 6 column. **f** Relative ATPase activities of p97 D2 and mutants. The activities are normalized to that of D2 WT. Data are shown as means ± SEM (*n* = 3 independent experiments). **g** Sequence alignment of the C-terminal region of human (Hs) p97 and *Saccharomyces cerevisiae* (Sc) Cdc48. **h** The substrate unfolding activity of Sc Cdc48 and the indicated mutants at 2.4 μM protomer concentration. Data are shown as means ± SEM (*n* = 3 independent experiments). AA, E315A/E588A, Cdc48 mutant deficient in ATPase activities of both D1 and D2 domains; SH, single hexamer; DH, double hexamer.

Since the two hexameric D2 rings mediate the formation of p97 DH, we examined the oligomerization status of p97 D2 in isolation using analytical size exclusion chromatography coupled to multi-angle laser light scattering (SEC-MALS). The ND1 (residues 1–462) and D1D2 (residues 210**–**806) fragments of p97 formed stable oligomers with or without different nucleotides (Supplementary Fig. S9a, b). In contrast, the isolated D2 domain wild-type (WT; residues 463–806) fractionated as multiple species during gel filtration chromatography (Fig. 6a). The relative ratios of these species varied in the presence of different nucleotides (ATP, ADP, and ATPγS), indicative of ATP-driven conformational dynamics.

We next introduced the E578A mutation to target the Walker B motif of the D2 ATPase. This mutation weakens ATP hydrolysis, but not ATP binding^23,35,45^. Without nucleotide or with ADP, D2 E578A fractionated as a single peak during gel filtration (Fig. 6b), suggesting that this mutation also weakens inter-subunit interactions in the D2 ring in the absence of nucleotides. The addition of ATP caused oligomerization of D2 E578A. SEC-MALS revealed a major peak of 240 kDa and a minor peak of 500 kDa for the ATP-bound D2 E578A oligomers (Fig. 6c, d), which likely corresponded to hexamer and double hexamers, respectively.

We tested whether the CTE mediated inter-subunit interactions in the isolated D2 ring. In contrast to D2 WT or E578A which could form multiple oligomeric species, D2 or D2 E578A with the CTE deleted (ΔCTE) existed exclusively as a monomer, with or without ATP (Fig. 6e; Supplementary Fig. S9c, d). Mutating the 3F motif in the CTE (3E; F768E/F771E/F773E) or its binding pocket in ɑ6 of the neighboring D2 (6A; L672A/F674A/L675A/M678A/N680A/F682A) also abolished oligomerization of D2 or D2 E578A with or without ATP (Fig. 6e; Supplementary Fig. S9c–e). This result further confirms the role of the CTE in strengthening the inter-subunit interactions of the D2 ring.

Both ɑ9 and the RD pair are critical structural elements that contribute to the dimerization of the SH. We next introduced mutations to disrupt the ɑ9–ɑ9 interface (4D; N750D/R753D/M757D/Q760D) or the RD pair (GG; R745G/D749G) in the isolated D2 domain. Unexpectedly, with or without ATP, the D2 GG and 4D mutants existed exclusively as monomers (Fig. 6e; Supplementary Fig. S9c, d). The GG mutation also disrupted the oligomerization of D2 E578A in the presence of ATP (Supplementary Fig. S9e). Thus, the SH dimerization interface in p97 DH is required for the oligomerization of D2 in isolation, including the formation of the single hexamer. This interesting result further suggests that, in addition to the tethering interactions involving the CTE, dimerization of the SH might help to strengthen the inter-subunit interactions in the D2 ring in each SH of the intact p97.

The isolated p97 D2 WT exhibited robust ATPase activity, while the D2 ATPase-deficient mutant E578A showed very weak activity (Supplementary Fig. S9f). D2 GG, 4D, ΔCTE, 3E, and 6A mutations, which disrupted D2 oligomerization, greatly decreased the ATPase activity of D2 (Fig. 6f). This finding suggests that the CTE and the SH dimerization interface are critical for the ATPase activity of D2.

### Requirement for ɑ9 and the CTE in substrate unfolding by Cdc48

We next tested whether the CTE and the SH dimerization interface were required for substrate unfolding by Cdc48. We deleted the CTE of Cdc48 (ΔCTE) and introduced mutations to Cdc48 to disrupt the ɑ9–ɑ9 interface (4D; N773D/R775D/A779D/Q782D) or the RD pair (GG; R767G/D771G) (Fig. 6g). Both DH and SH of Cdc48 WT unfolded the model substrate mEos3.2 efficiently (Fig. 6h). Strikingly, both SH and DH conformers of Cdc48 ΔCTE and GG were inactive in substrate unfolding, and the SH conformer of Cdc48 4D, which could not form the DH, was also inactive (Fig. 6h). Thus, the CTE and the dimerization interface are both critical for substrate unfolding.

## Discussion

The p97 DH clearly exists in vitro and likely exists in living cells. We show here that the p97 DH is an active ATPase and Cdc48 DH is an active unfoldase. The cryo-EM structures of p97 DH captures an ATPase-active state of p97 with ordered pore loops and reveal a novel interaction between the CTE and a hydrophobic cleft in the neighboring protomer in the D2 ring. This newly observed CTE interaction is required for the ATPase activity of p97 and substrate unfolding by Cdc48. Surprisingly, we also show that the SH dimerization interface, including α9 and the RD pair of p97/Cdc48, is required for oligomerization of isolated D2 domains and for substrate unfolding, indicating the functional involvement of this surface in the catalytic cycle of p97/Cdc48.

The functional requirement of the CTE in substrate unfolding of p97/Cdc48 likely stems from its ability to strengthen the D2 ring and to maintain subunit coupling within the D2 ring during ATP hydrolysis. The basis for the requirement of the dimerization interface in the activity of p97/Cdc48 is less clear. We envision two possibilities. In the first possibility, the DH is an obligatory intermediate in the catalytic cycle of p97/Cdc48. The SH dimerization strengthens the D2 ring and organizes the D2 ATPase into a potentiated state with ordered pore loops, which is poised to unfold substrate. Upon substrate engagement, the DH dissociates to allow the pore loops to adopt the staircase configuration for the initial unfolding of the ubiquitin. The DH may only be required for the processing of a certain subset of substrates. In the second possibility, the DH is not an obligatory intermediate during substrate unfolding. Only the SH can unfold substrate and the DH represents a resting state that is incapable of substrate unfolding. ATP hydrolysis of the D2 ring dissociates the DH into two SHs, which is the active conformer that unfolds substrates. The dimerization interface interacts with segments of the unfolded ubiquitin or substrate to promote the translocation of the unfolded protein chains through the central pore. Future structural and crosslinking studies are needed to distinguish between these two possibilities.

p97/Cdc48 is involved in a variety of cellular events. It collaborates with diverse cofactors and adaptors to deal with different types of substrates^1,2,5,11,28,46,47^. Some of these cofactors bind to the C-terminal segment that follows the structurally resolved CTE of p97/Cdc48. It will be interesting to test whether the binding of these cofactors regulates the formation of the DH and vice versa.

In conclusion, our study reveals a functional requirement of the C-terminal extension of p97/Cdc48 and the SH dimerization interface in substrate processing. Future studies aimed at elucidating the structural basis of this requirement will deepen the mechanistic understanding of this fascinating ATPase machine.

## Materials and methods

### Protein expression and purification

cDNAs encoding full-length human p97 (residues 1–806; P55072 in Swiss-UniProt) were amplified by PCR and cloned into the pET28a and pET21b vectors (Novagen) to produce fusion proteins with N-terminal His_6_-tag and C-terminal His_6_-tag, respectively. The cDNAs for human SPRTN (residues 1–270) and p97 D2 domains (residues 463–806) were cloned into the pGEX-6p-1 vector (Novagen) to produce fusion proteins with an N-terminal GST tag. Full-length SPRTN was cloned into the pMal-p5 vector (New England Biolabs) to produce a fusion protein with an N-terminal MBP tag. The cDNA for the budding yeast Cdc48 (residues 1–83; P25694 in Swiss-UniProt) was cloned into the pET21b vector. Mutants of p97, Cdc48, and SPRTN were generated using PCR-mediated site-directed mutagenesis. All constructs were verified by DNA sequencing.

For the expression of recombinant human p97 and yeast Cdc48 proteins, BL21 (DE3) *Escherichia coli* cells containing the desired plasmid were grown in Teffic Broth (TB) medium containing ampicillin (100 μg/L for pET21b and pGEX-6p-1 plasmids) or kanamycin (50 μg/L for the pET28a plasmid) with shaking at 37 °C to OD600 of 1.0. The cell culture was cooled to 18 °C, induced with 0.5 mM IPTG for 12–15 h, and harvested by centrifugation. The cell pellet from each 500 mL culture was suspended in 50 mL of lysis buffer (25 mM Tris-HCl pH 7.5, 300 mM NaCl, 5 mM MgCl_2_, 5 mM β-mercaptoethanol, and 1 mM PMSF, with 10 mM imidazole added for His_6_-tagged proteins). The cells were lysed by sonication or a Microfluid cell disruptor. The lysate was centrifuged at 40,000 × *g* for 50 min at 4 °C, and the resulting supernatant was incubated with pre-equilibrated Ni^2+^-NTA agarose beads for His_6_-tagged proteins (Qiagen) or Glutathione Sepharose 4B beads for GST-tagged proteins (GE Healthcare) for 2 h at 4 °C. The beads were washed with 100 mL wash buffer (25 mM Tris-HCl pH 7.5, 300 mM NaCl, 5 mM MgCl_2_, 5 mM β-mercaptoethanol, with 20 mM imidazole added for His_6_-tagged proteins). The His_6_-tagged proteins were eluted with 15 mL elution buffer (25 mM Tris-HCl pH 7.5, 150 mM NaCl, 5 mM MgCl_2_, 5 mM β-mercaptoethanol, and 300 mM imidazole). The GST-tagged proteins were incubated with HRV 3C protease overnight. The His_6_-tagged protein was loaded onto Resource Q column and fractionated by AKTA Pure system (GE Healthcare). The pooled peak fractions were combined and concentrated with an Amicon Ultra-15 centrifugal filter unit (NMWL = 100 kDa for p97 and Cdc48). For the p97 D2 domain, following the HRV 3C protease cleavage, the mixture was eluted and concentrated with an Amicon Ultra-15 centrifugal filter unit (NMWL = 30 kDa). For all concentrated proteins, the mixture was then fractionated in elution buffer (25 mM HEPES-NaOH pH 7.5, 100 mM NaCl, 5 mM MgCl_2_, 0.5 mM TCEP) with a gel filtration column (Superose 6 Increase 10/300 GL; GE Healthcare). Fractions that contained p97 or Cdc48 were collected, analyzed by 10% SDS-PAGE, aliquoted, and stored at –80 °C.

### Electron microscopy

Freshly purified p97 or Cdc48 (5 µL, 30 μg/mL) were incubated with 1 mM ATP or ATPγS for 1 h before being applied to glow-discharged EM grids (Formvar/Carbon 200 Mesh; Electron Microscopy Sciences). After 30 s, excess sample was blotted, and the grid was washed once with sample buffer and stained with 2% (w/v) uranyl acetate (UA) or uranyl formate (UF) for 60 s. Excess staining solution was blotted with filter paper, and the grids were air-dried before being transferred into a Tecnai T12 electron microscope (FEI) operated at 120 kV. Images were recorded with a Gatan 4k × 4k CCD camera at a nominal magnification of 30,000×. Defocuses were set at about −2 μm.

Human p97–SPRTN complex was incubated with 1 mM ATPγS for 1 h before being applied to glow-discharged Quantifoil R1.2/1.3 300-mesh gold holey carbon grid (Ted Pella). 3 µL sample was applied and the grids were blotted for 3.0 s under 100% humidity at 4 °C before being plunged into liquid ethane using a Mark IV Vitrobot (FEI). Micrographs were acquired on a Titan Krios microscope (FEI) operated at 300 kV with a K2 Summit direct electron detector (Gatan), using a slit width of 20 eV on a GIF-Quantum energy filter. Images were recorded with the EPU software (FEI) in super-resolution counting mode with a super resolution pixel size of 0.54 Å. The defocus range was set from −0.9 to −2.4 μm. Each micrograph was dose-fractionated to 40 frames under a dose rate of 5 e^−^ per pixel per second, with a total exposure time of 12 s, resulting in a total dose of about 52 e^−^/Å^2^.

### Image processing

Micrographs were motion corrected with MotionCor2^48^. The CTF parameters of the micrographs were estimated using the GCTF program^49^. All other steps of image processing were performed using RELION^50,51^. Initially, about 1000 particles were manually picked from a few micrographs. Class averages representing projections of p97 in different orientations were selected from the 2D classification of the manually picked particles and used as templates for automatic particle picking from the full dataset. 692,435 particles in total were picked from 2246 micrographs. The particles were extracted and subjected to 2D classification. After bad particles were discarded, a total of 613,892 particles were selected for 3D classification using the 15 Å low-pass filtered p97 structure (5FTJ) as the initial model. Two of the 3D classes showed good secondary structure features and were selected for further second round 3D classification. 313,356 particles from class 4 (representing p97 single hexamers) were selected and subjected to 3D classification (4 classes). All 4 classes showed good secondary structure features. Two classes showing similar features were combined. After 3D refinement with C6 symmetry imposed and CTF refinement by RELION 3, all resulting 3D reconstructions from class 1 (79,221 particles), class 2 (65,108 particles), and class 3 (65,527 particles) showed clear six-fold symmetry with resolutions of 3.21, 3.24, 3.30 Å, respectively. 70,885 particles from class 1 (representing p97 double hexamer) of the first round 3D classification were selected and subjected to 3D classification (3 classes). All 3 classes showed good secondary structure features. Two classes that showed highly similar features were combined. After 3D refinement with D6 symmetry imposed and CTF refinement by RELION 3, the resulting 3D reconstructions from class 1 (20,139 particles) and class 2 (50,746 particles) showed clear six-fold reflection symmetry and two-fold rotation symmetry with resolutions of 3.15 and 3.32 Å, respectively. All resolutions were estimated by applying a soft mask around the protein density and the gold-standard Fourier shell correlation (FSC) = 0.143 criterion. ResMap was used to calculate the local resolution map^37^.

### Atomic model building and refinement

The X-ray structure of human p97 (PDB code: 3CF3; chain A) was used as the starting model and docked into the EM maps with UCSF Chimera^52^. The models were manually adjusted and built in Coot^53^ and then refined against summed maps using phenix.real_space_refine implemented in PHENIX^54^. The fitting of the model to the EM map was examined and refined through reciprocal refinement using Coot and Phenix. Symmetry (C6 for p97 single hexamer and D6 for p97 double hexamer) was imposed during refinement steps at the end. The resulting atomic model was further examined in Coot and refined in reciprocal space by Phenix. FSC values were calculated between the resulting model and the two half-maps, as well as the averaged map of the two half-maps. The quality of the models was evaluated with MolProbity^55^ and EMRinger^56^. All figures were prepared with PyMOL^57^ or Chimera^52^.

Because our EM maps had resolutions of about 3.3 Å, we could confidently model the nucleotides states in our DHs. We defined the two conformations of p97 DH and bound nucleotides as follows: DH conformer I (D1-ATPγS and D2-ADP; PDB code:7VCU) and DH conformer II (D1-ATPγS and D2-ATPγS; PDB code: 7VCS). These two conformers were similar to the deposited structures of p97 dodecamer (PDB code: 7K56 and 7K57) at lower resolutions^43^, which did not define bound nucleotides. The three SHs were defined as follows: SH conformer I (D1-ATPγS and D2-ATPγS; PDB code:7VCV), SH conformer II (D1-ATPγS and D2-ATPγS; PDB code:7VCX), SH conformer III (D1-ATPγS and D2-ADP; PDB code:7VCT). These three conformers corresponded to the three p97 conformers (PDB code: 5FTK, 5FTL, and 5FTM) reported previously^20^, except that the bound nucleotides were different.

### ATPase assay

The ATPase reactions were performed in white OptiPlate-384 plates (Perkin Elmer) at room temperature using the ADP-Glo Kinase Assay kit (Promega). p97, Cdc48, and their mutants (120 nM monomer concentration) were diluted in the Reaction Buffer (25 mM Tris-HCl pH 7.5, 100 mM NaCl, 20 mM MgCl_2_, 1 mM DTT) and incubated at room temperature for 15 min. p97 cofactors (the SPRTN SHP and Ubxd1 VIM peptides; synthesized by KareBay Biochem) were added and incubated at room temperature for 30 min. ATP was added to a final concentration of 0.2 mM in reactions each with a final volume of 10 µL and incubated for 60 min. 10 µL ADP-Glo Reagent was added to the reaction and incubated for 30 min at room temperature. 20 µL detect reagent from the kit was added to the reaction, and incubated for 30 min. Luminescence was measured with the VICTOR 3 V Multilabel Plate Reader (Perkin Elmer). Standard curve was prepared according to the manufacturer’s instructions to calculate the conversion of ATP to ADP based on luminescence. All data were normalized to the reading of the sample containing the Reaction Buffer alone with no proteins. All ATPase assays were repeated three times.

### Gel filtration assay

For p97 D2 domain WT and mutants, 500 µL purified proteins (0.5–1.0 mg/mL) alone or preincubated with 1 mM ATP or different nucleotides were loaded onto the Superose 6 Increase 10/300 GL column pre-equilibrated with elution buffer (25 mM HEPES-NaOH pH 7.5, 100 mM NaCl, 5 mM MgCl_2_, 0.5 mM TCEP). Fractions that contained p97 D2 were collected and analyzed by 10% SDS-PAGE.

### SEC-MALS assay

SEC-MALS assay for p97 D2 domain (E578A) with or without 1 mM ATP was performed using a Jasco PU-2080 Plus system consisting of a pump, a vacuum degasser, an autosampler, and a Silica Gel KW804 column (Shodex). Detection was performed using a triple-angle light scattering detector (Mini- DAWN™ TREOS, Wyatt Technology), a Shimadzu refractometer (RID-10A), and a SpectraSeries UV100 detector. The size exclusion column (Superdex 200 Increase 10/300 GL; GE Healthcare) used for the SEC-MALS analysis was equilibrated with the SEC buffer (25 mM HEPES-NaOH pH 7.5, 50 mM NaCl, 5 mM MgCl_2_, 0.5 mM TCEP). 100 μl protein (~1 mg/mL) was injected. The intrinsic instrument base line for each data channel was subtracted. The molar mass of the elution profile was determined with the ASTRA software (Wyatt Technology).

### Substrate unfolding assay

Yeast Ufd1/Npl4 and the substrate mEos3.2 for the unfolding assay were prepared as described^7,29^. The unfolding assays for yeast Cdc48 were performed as described^7,29^. Different concentrations of Cdc48 or its mutants, 400 nM Ufd1/Npl4 and 400 nM polyubiquitinated mEos3.2 were incubated in the reaction buffer (40 mM HEPES-NaOH pH 7.5, 100 mM NaCl, 10 mM MgCl_2_, 0.5 mg/mL protease-free BSA) for 10 min before the addition of 2 mM the ATP regeneration mix (2 mM ATP, 20 mM phosphocreatine, 100 μg/mL creatine kinase). Fluorescence of mEos3.2 (excitation: 540 nm; emission: 580 nm) was measured at 30 s intervals in an M5 plate reader (Spectramax) for 30 min. Fluorescence values were normalized by subtracting the measured fluorescence of polyubiquitinated mEos3.2 denatured in 6 M Guanidine-HCl. All unfolding assays were repeated three times.

### Statistical analysis

When indicated, the mean and standard error of the mean of three independent experiments were presented. No statistical methods were used to predetermine sample size or applied to data analysis. The experiments were not randomized. The investigators were not blinded to allocation during experiments and outcome assessment.

## Supplementary information

Supplementary Information

## Data Availability

The cryo-EM density maps of the p97 have been deposited to the Electron Microscopy Data Bank under the accession numbers EMD-31896 (DH conformer I), EMD-31894 (DH conformer II), EMD-31897 (SH conformer I), EMD-31899 (SH conformer II), and EMD-31895 (SH conformer III). Atomic coordinates have been deposited to the RCSB Protein Data Bank under the accession numbers 7VCU (DH conformer I), 7VCS (DH conformer II), 7VCV (SH conformer I), 7VCX (SH conformer II), 7VCT (SH conformer III).
