# Supplementary material for: Cryo-EM structures of human p97 double hexamer capture potentiated ATPase-competent state
Source: Cell Discov. 2022 Feb 22;8:19. doi: 10.1038/s41421-022-00379-1 (PMC8861141; doi:10.1038/s41421-022-00379-1)
Supplement: Supplementary file 1 — Supplementary Information [file 41421_2022_379_MOESM1_ESM.pdf]

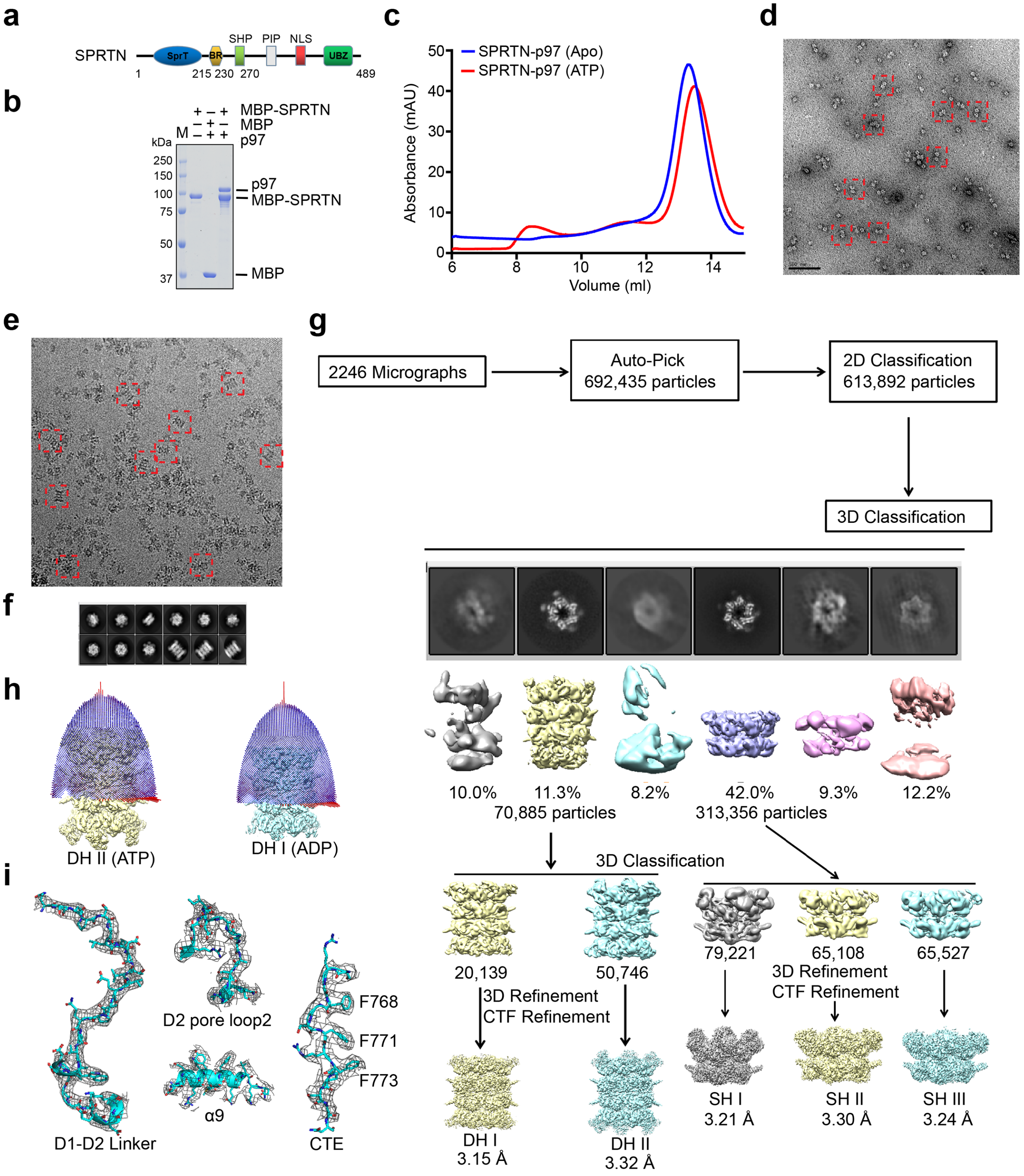

Fig. S1 EM analysis of human p97-SPRTN complex. a Domains and motifs of human SPRTN. **b** Binding between SPRTN and p97. Beads bound to MBP or MBP-SPRTN were incubated with p97 and washed. Proteins bound to beads were analyzed by SDS-PAGE and stained with Coomassie Brilliant Blue. **c** The gel filtration profiles of the p97-SPRTN complex with or without ATP on the Superose 6 column. **d** Negative-stain EM micrograph of the p97-SPRTN complex. Particles likely represent p97 double hexamers (DH) are boxed with red dashed lines. **e**

Representative raw cryo-EM micrograph of the p97-SPRTN complex after motion correction by RELION. The DH particles in side views are boxed with red dashed lines. **f** Representative 2D class averages. **g** Workflow of cryo-EM data processing to obtain the 3D reconstructions of p97 single hexamer (SH) and DH. **h** Distribution of particle orientations in the last round of structural refinement of DH conformers I (right) and II (left). **i** Representative cryo-EM density segments of the EM map highlighting key structural elements of p97 DH with a contour level at 0.020 (5σ).

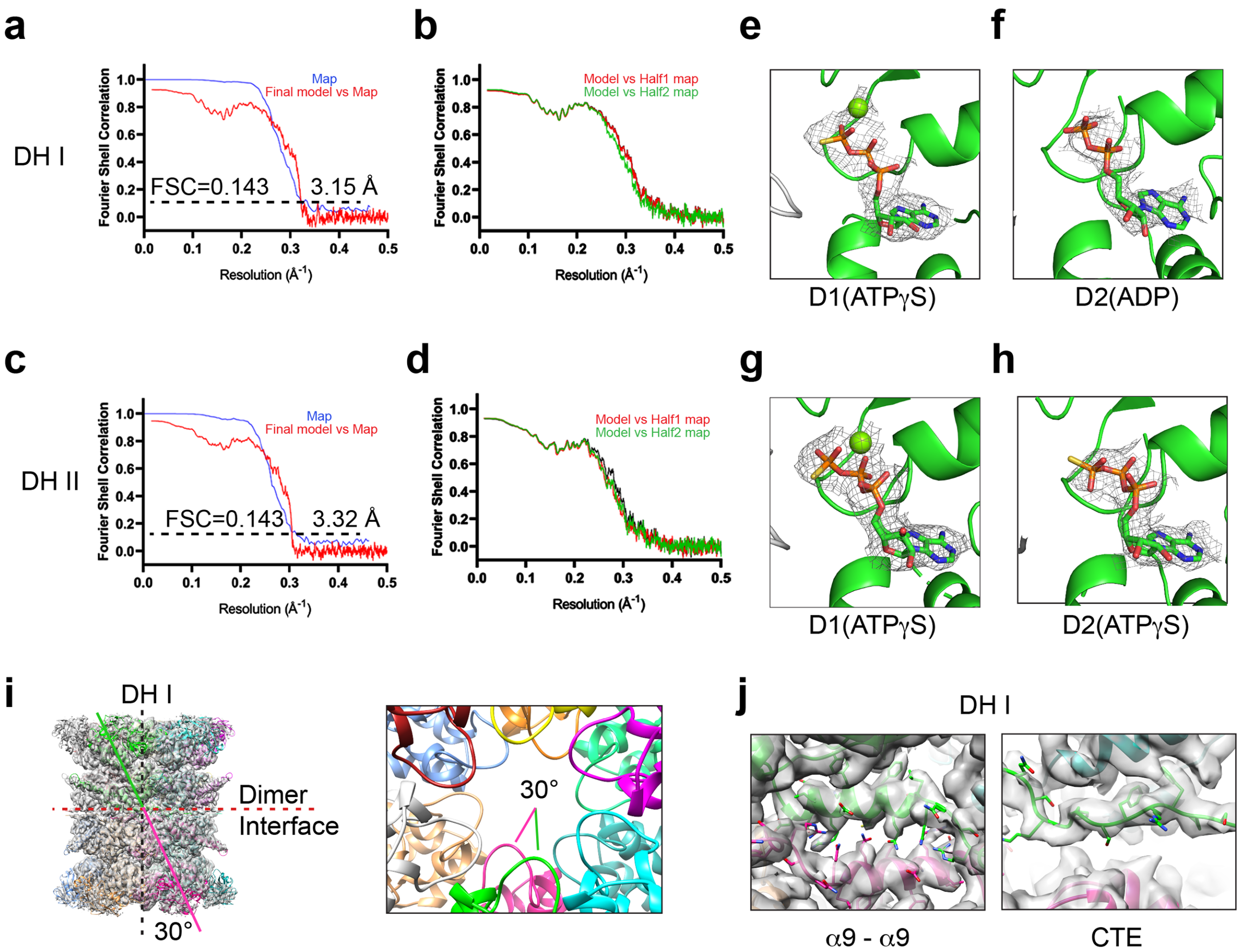

Fig. S2 Cryo-EM density of the bound nucleotides in p97 DHs. **a** The gold-standard Fourier shell correlation (FSC) curves for the overall cryo-EM map (blue) and the final refined model versus map (red) of p97 DH conformer I. **b** FSC curves for model versus half map 1 (working, red) or half map 2 (free, green) of p97 DH conformer I. **c** The gold-standard FSC curve for the overall cryo-EM map (blue) and the final refined model versus map (red) of p97 DH conformer II. **d** FSC curves for the model versus half map 1 (working, red) or half map 2 (free, green) and the model versus final refined map (black) of p97 DH conformer II. **e** ATPγS overlaid with its EM density in the D1 ring of DH conformer I. The contour levels in this figure are set at 0.020 (5σ). **f** ADP overlaid with its EM density in the D2 ring of DH conformer I. **g** ATPγS overlaid with its EM density in the D1 ring of DH conformer II. **h** ATPγS overlaid with its EM density in the D2 ring of DH conformer II. **i** p97 DH I fitted into EM maps (sideview; left) and ribbon diagram of the D2 ring pore loops (top view; right). The angles between the two interacting protomers from the two SHs along the central axis are indicated. **j** The DH interface and the C-terminal extension (CTE) of DH I fitted into the EM map.

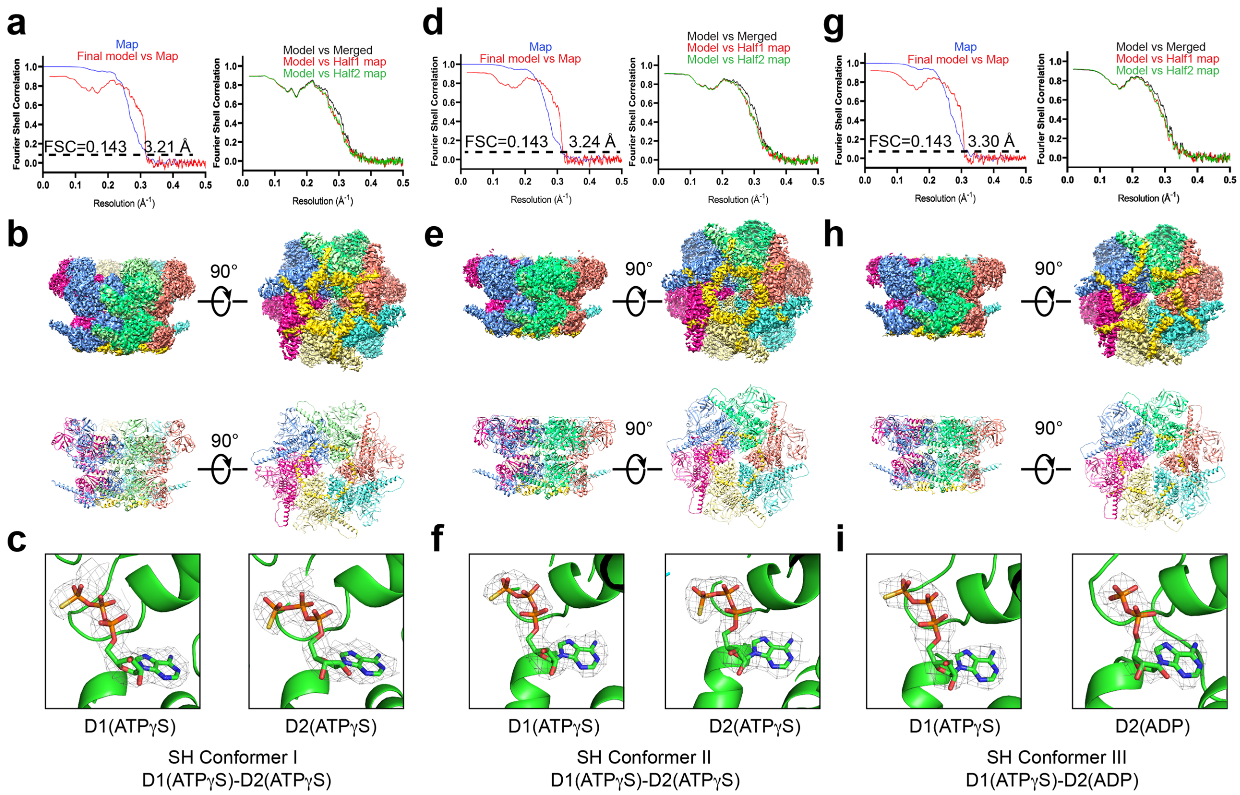

Fig. S3 Cryo-EM maps and models of p97 single hexamers (SHs). a (Left panel) The gold-standard FSC curves (left) for the overall cryo-EM map (blue) and final refined model (red) of p97 SH conformer I. (Right panel) FSC curves for the model versus half map 1 (working, red) or half map 2 (free, green) and the model versus final refined map (black) of p97 SH conformer I. **b** The cryo-EM map and final model for conformer I with each protomer colored differently. **c** The cryo-EM density of the nucleotides in the D1 and D2 domains of p97 SH conformer I. The contour levels in this figure are set at 0.010 (2σ). **d** (Left panel) The gold-standard FSC curves (left) for the overall cryo-EM map (blue) and final refined model (red) of p97 SH conformer II. (Right panel) FSC curves for the model versus half map 1 (working, red) or half map 2 (free, green) and the model versus final refined map (black) of p97 SH conformer II. **e** The cryo-EM map and final model for conformer II with each protomer colored differently. **f** The cryo-EM density of the nucleotides in the D1 and D2 domains of p97 SH conformer II. **g** (Left panel) The gold-standard FSC curves (left) for the overall cryo-EM map (blue) and final refined model (red) of p97 SH conformer III. (Right panel) FSC curves for the model versus half map 1 (working, red) or half map 2 (free, green) and the model versus final refined map (black) of p97 SH conformer III. **h** The cryo-EM map and final model for conformer III with each protomer colored differently. **i** The cryo-EM density of the nucleotides in the D1 and D2 domains of p97 SH conformer III.

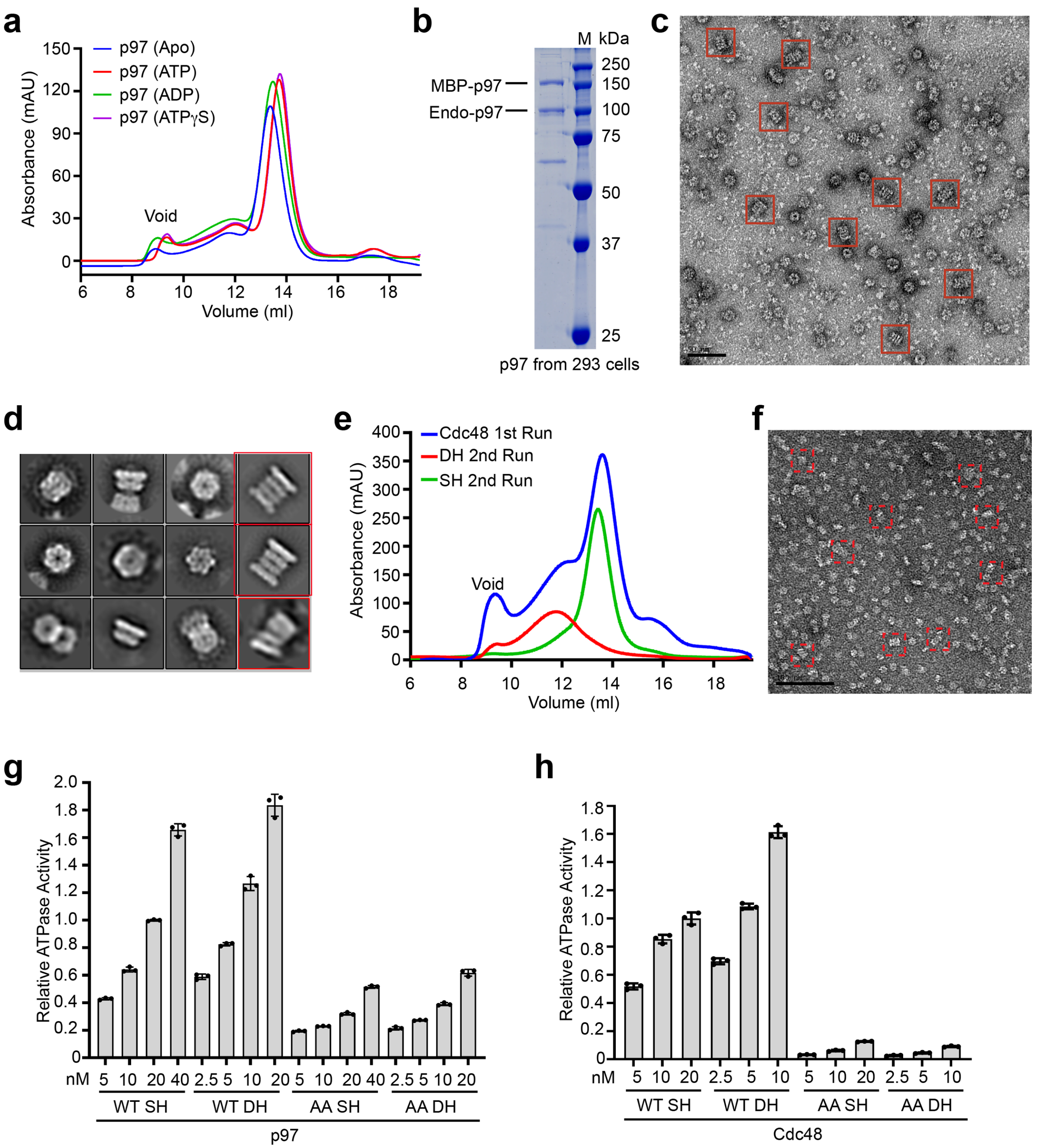

Fig. S4 Both human p97 and yeast Cdc48 can form double hexamers (DHs). a Gel filtration profiles of human p97 without nucleotides (Apo) or with ATP, ADP or ATPγS on the Superose 6 column. **b** SDS-PAGE of recombinant human p97 expressed in HEK293 cells using the BacMam system. **c** Representative negative-stain EM micrograph of human p97 purified from HEK293 cells. DH particles in side views are boxed with red dashed lines. **d** Representative 2D class averages of cryo-EM data for p97 purified from 293 cells. DH classes in side views are boxed by red squares. **e** Gel filtration profiles of Cdc48 with ATP on the Superose 6 column. **f** Negative-stain EM micrograph of Cdc48. DH particles in side views are boxed with red dashed lines. **g** Relative ATPase activities of human p97 single hexamer (SH) and DH at different concentrations. The final

concentrations of SH and DH (instead of the protomer concentrations) are indicated below the graph. The activities are normalized to that of WT SH at 20 nM concentration. WT, wild type; AA, E305A/E578A, p97 mutant deficient in ATPase activities of both D1 and D2 domains. Mean ± SEM (n = 3 independent experiments). **h** Relative ATPase activities of Sc Cdc48 SH and DH at different concentrations. The final concentrations of SH and DH (instead of the protomer concentrations) are indicated below the graph. The activities are normalized to that of WT SH 20 nM concentration. WT, wild type; AA, E315A/E588A, Cdc48 mutant deficient in ATPase activities of both D1 and D2 domains. Mean ± SEM (n = 3 independent experiments).

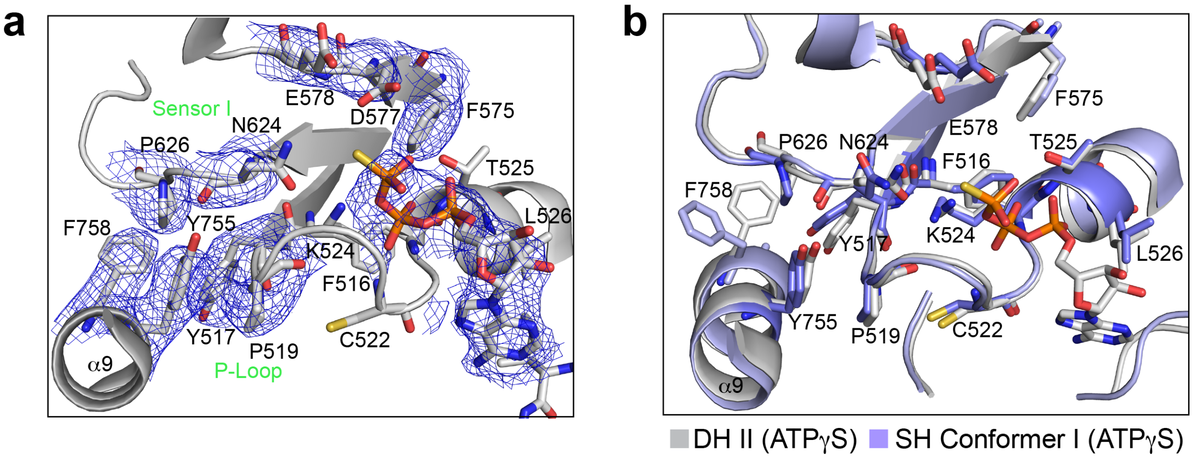

Fig. S5 The α9 helix of p97 stabilizes the D2 ATP-binding pocket. a The Cryo-EM density of the α9 helix and D2 ATP-binding pocket in p97 DH II. The contour level is set at 0.020 (5σ). **b** Structural comparison of the α9 helix and D2 ATP-binding pocket in DH conformer II and SH conformer I.

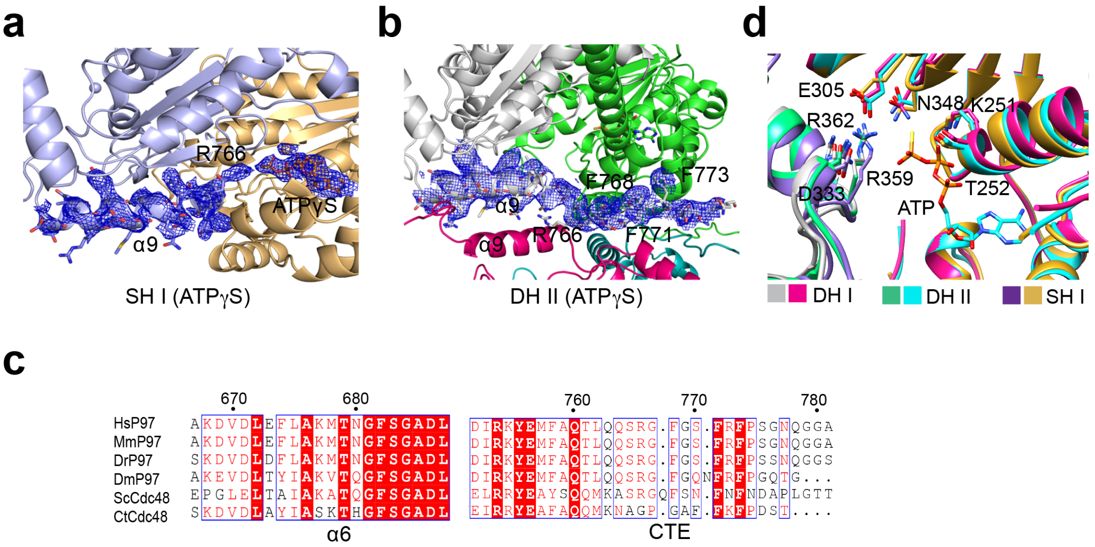

Fig. S6 Interactions involving the C-terminal extension (CTE) of p97 single hexamer (SH) and double hexamer (DH). a The cryo-EM density of α9 and R766 in the SH conformer I. **b** The cryo-EM density of α9 and CTE in the DH conformer II. The contour levels for the SH and DH are set at 0.010 (2σ) and 0.020 (5σ), respectively. **c** Sequence alignment of the CTE-binding α6 helix (left) and the CTE (right). The sequences were aligned using Clustal Omega and numbered according to human p97. **d** Superimposition of the ATP-binding pocket of the D1 domain from p97 SH I and DH I and II. Color schemes for each conformer are indicated below.

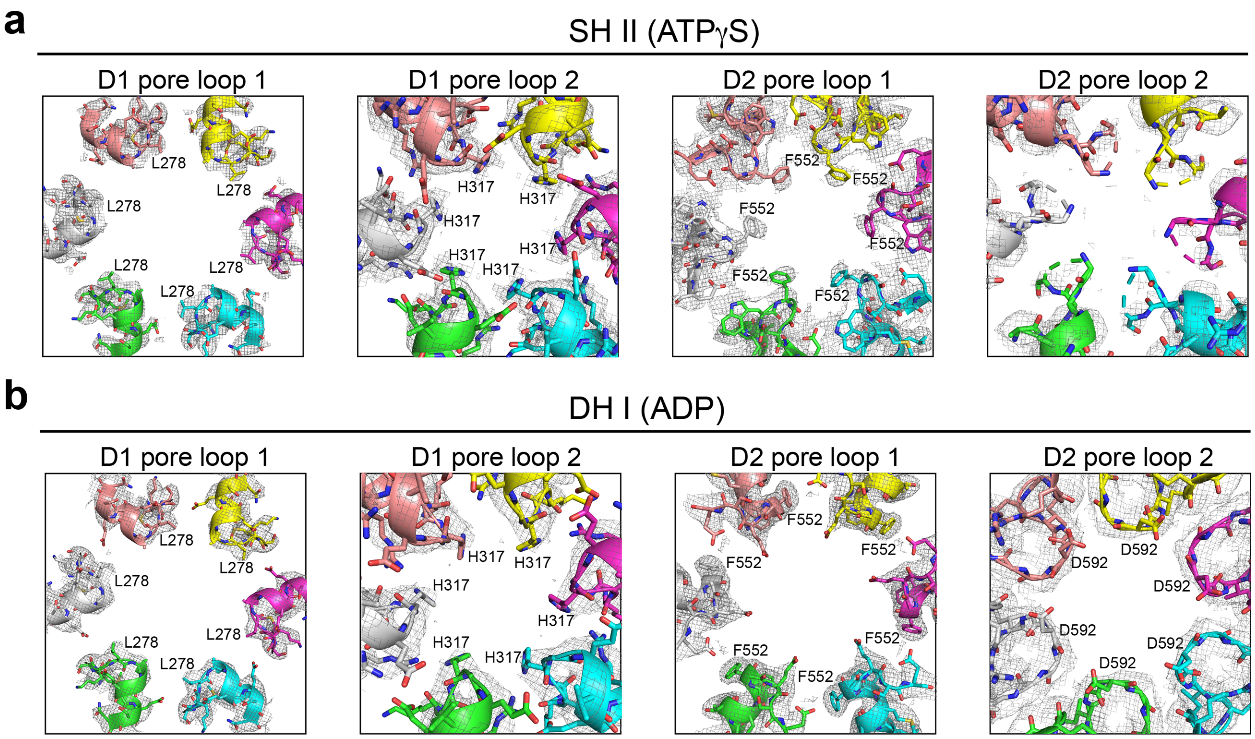

Fig. S7 The pore loops are better defined in the p97 double hexamer (DH). a,b Cryo-EM density of the pore loops in the D1 and D2 rings of p97 SH II (**a**) and DH I (**b**). The contour levels for the SH and DH are set at 0.010 (2σ) and 0.020 (5σ), respectively.

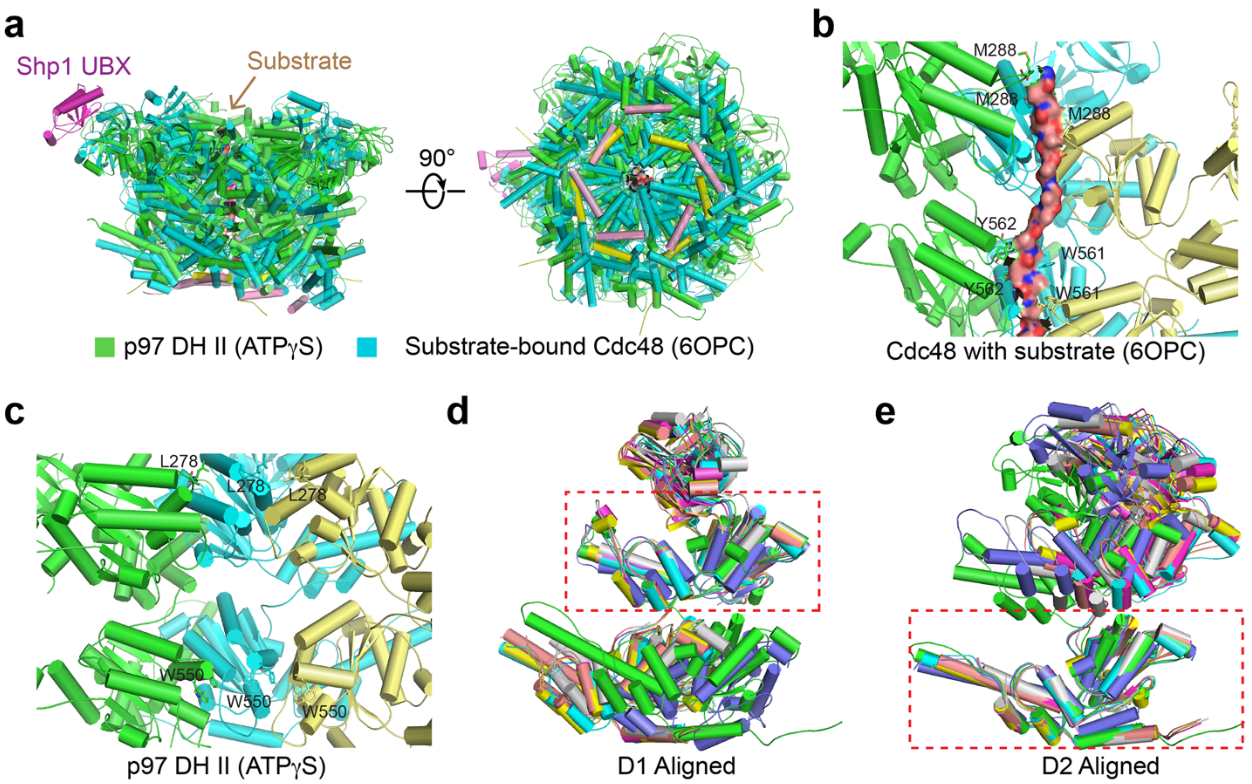

Fig. S8 Structural comparison of p97 DH and substrate-bound Cdc48. a The side (left) and bottom (right) views of the superimposed cartoon diagrams of p97 DH conformer II (green) and substrate-bound Cdc48 (cyan; PDB code: 6OPC). The Sc Shp1 UBX domain is colored magenta. α9 helices of p97 and Cdc48 are colored yellow and light purple, respectively. The substrate is shown in surface drawing and colored by elements. **b** The staircase distribution of the D1 and D2 pore loops in substrate-bound Cdc48. A, B, C subunits are colored green, cyan and yellow, respectively. The substrate is shown in surface drawing and colored by elements. **c** The symmetric and planar distribution of pore loops in the substrate-free conformation of p97 DH II. A, B, C subunits are colored green, cyan and yellow, respectively. **d** Superimposed cartoon diagrams of a single p97 protomer (green) and all six protomers (each colored differently) of Cdc48 (PDB code: 6OPC), with their D1 domain aligned and boxed with a red rectangle. **e** Superimposed cartoon diagrams of a single p97 protomer (green) and all six protomers (each colored differently) of Cdc48 (PDB code: 6OPC), with their D2 domain aligned and boxed with a red rectangle.

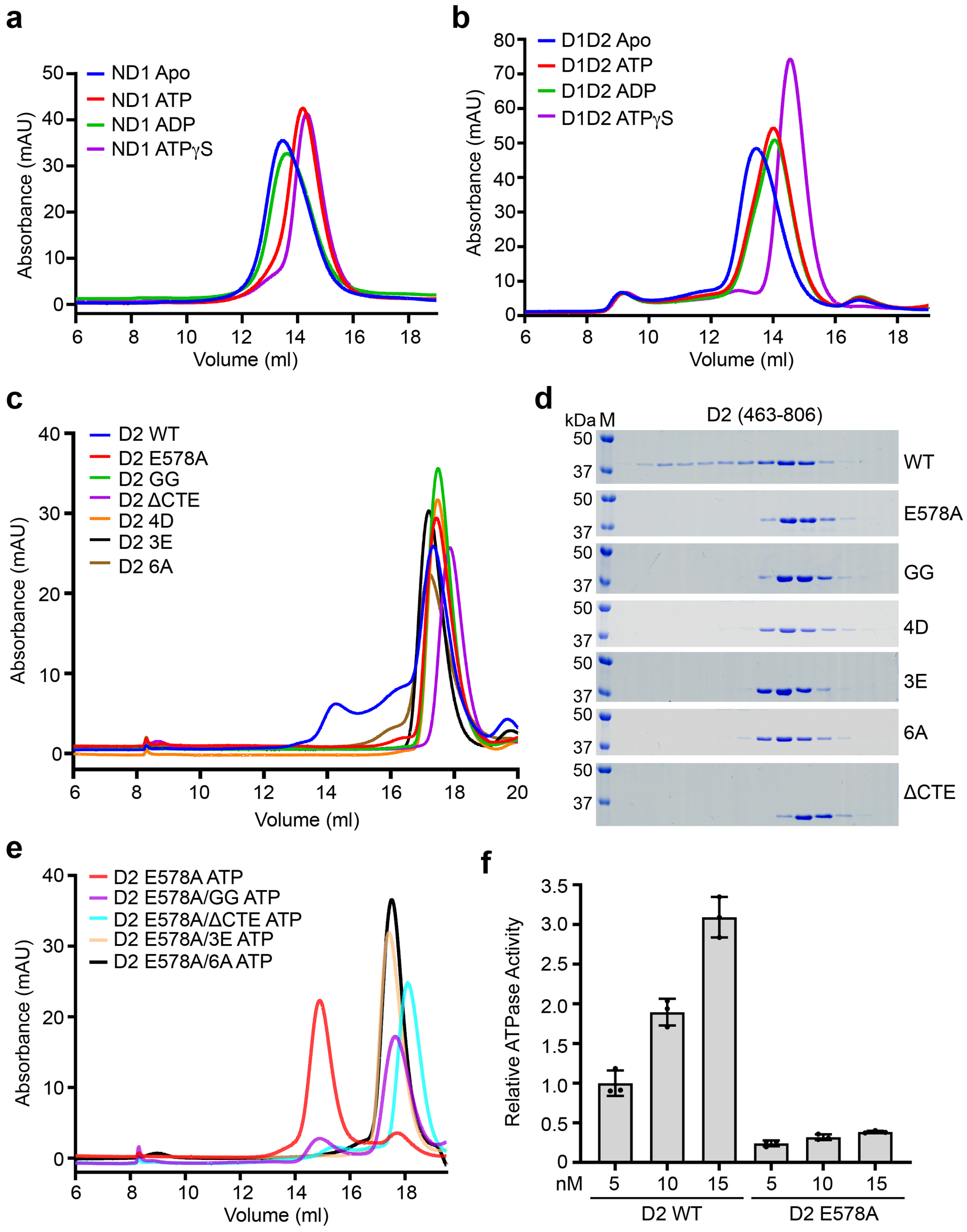

Fig. S9 The C-terminal extension and α9 strengthen the D2 ring. a Gel filtration profiles of the p97 ND1 fragment (residues 1-462) without (Apo) or with different nucleotides on the Superose 6 column. **b** Gel filtration profiles of the p97 D1D2 fragment (residues 210-806) without (Apo) or

with different nucleotides on the Superose 6 column. **c** Gel filtration profiles of p97 D2 (residues 463-806) wild type (WT) and the indicated mutants without nucleotides on the Superose 6 column. **d** Coomassie stained SDS-PAGE of p97 D2 WT and mutants from gel filtration fractions in (**c**). **e** Gel filtration profiles of p97 D2 E578A and its mutants with ATP on the Superose 6 column. **f** Relative ATPase activities of p97 D2 WT and E578A at indicated concentrations. The activities are normalized to that of D2 WT at 5 nM concentration. Mean ± SEM (n = 3 independent experiments).

Supplementary Table S1. Cryo-EM statistics and model refinement for p97 DH and SH

	DH I	DH II	SH I	SH II	SH III
**Data collection and processing**					
Microscope	FEI Titan Krios				
Detector	Gatan K2 Summit, 300 kV				
Magnification	105, 000 ×				
Pixel size (Å)	1.08				
Total electron dose (e^-/Å^2)	52				
Exposure rate (e^-/pixel/sec)	5.125				
Defocus range (μm)	-0.9 – -2.4				
Micrographs collected	2350				
Micrographs used	2246				
**Reconstruction**					
Softwares	RELION 2.1, RELION 3.0				
Total extracted particles	692,435				
Particles number for reconstruction	20,139	50,746	79,221	65,108	65,527
Particles number for refinement	20,139	50,746	79,221	65,108	65,527
Symmetry imposed	D6	D6	C6	C6	C6
Map resolution (Å) (FSC 0.143)	3.15	3.32	3.21	3.24	3.30
Map sharpening B-factor (Å^2)	-81.66	-67.32	-115.33	-114.08	-109.82
**Refinement**					
Softwares	Coot, phenix.real_space_refine in the PHENIX package				
Model resolution (Å)	3.1	3.3	3.2	3.2	3.3
Model composition					
Number of protein atoms	71,244	72,072	35,652	35,106	35,340
Number of ligand atoms	708	756	384	384	354
B factors (Å)					
Protein	127.9	128.1	88.9	67.8	81.5
Ligand	94.8	93.2	43.4	25.9	44.7
R.M.S deviations					
Bonds lengths (Å)	0.006	0.008	0.012	0.016	0.007
Bonds angles (°)	1.12	1.13	1.31	1.43	1.18
**Validation**					
MolProbity score	1.55	1.59	1.59	1.47	1.45
EMRinger score	2.00	1.94	2.50	2.62	2.62
All-atom clashscore	4.93	4.79	3.88	2.86	3.53
Rotamer outliers (%)	0	0	0	0	0
C-beta deviations	0	0	0	0	0
Ramachandran plot statistics					
Preferred (%)	95.76	95.00	93.78	94.16	95.64
Allowed (%)	4.24	5.00	6.22	5.84	4.36
Outlier (%)	0	0	0	0	0
